# Artificial intelligence and oral microbiome: Reshaping the diagnostic and therapeutic paradigm of OSCC

**DOI:** 10.1002/ctm2.70723

**Published:** 2026-07-05

**Authors:** Rui Shi, Yuan Zhi, Ling Gao, Shao‐Ming Li, Ke‐Qian Zhi, Wen‐Hao Ren

**Affiliations:** ^1^ Department of Oral and Maxillofacial Reconstruction the Affiliated Hospital of Qingdao University Qingdao China; ^2^ School of Stomatology, Qingdao University Qingdao China; ^3^ Department of Oral and Maxillofacial Surgery the Affiliated Hospital of Qingdao University Qingdao China; ^4^ Department of Oral and Maxillofacial Surgery Peking University School and Hospital of Stomatology Beijing China; ^5^ Key Lab of Oral Clinical Medicine The Affiliated Hospital of Qingdao University Qingdao Shandong China

**Keywords:** artificial intelligence, diagnosis, machine learning, oral microbiome, oral squamous cell carcinoma (OSCC), prognosis, tumour microenvironment

## Abstract

**Background:**

Oral squamous cell carcinoma (OSCC) remains a major clinical challenge, with delayed diagnosis, frequent resistance to therapy, and poor long‐term survival.

**Methods:**

This review systematically evaluates the methodological framework for applying AI to oral microbiome data in OSCC. Emerging paradigms, including self‐supervised learning for leveraging unlabelled data and explainable AI (XAI) techniques for model interpretability, are also discussed. Model evaluation relies on cross‐validation, hyperparameter optimisation, and performance metrics such as AUC, accuracy, sensitivity, specificity, and F1‐score.

**Results:**

Multiple studies demonstrate that AI‐based classifiers, especially random forest models built on salivary or tissue‐derived microbial features, achieve outstanding discrimination between OSCC patients and healthy controls in retrospective, single‐centre cohorts, with reported AUC values exceeding 0.99 and accuracy >95%. However, these exceptional metrics should be interpreted with caution, as they are susceptible to cohort size, sampling site heterogeneity, batch effects, feature‐selection bias, and the absence of independent external validation. Beyond binary diagnosis, AI has been successfully applied to predict lymph node metastasis, explore tumour metabolic reprogramming, and assess environmental interactions. Integrated multi‐omics approaches further enhance the specificity and clinical relevance of microbial biomarkers.

**Conclusions:**

The convergence of AI and oral microbiome analysis is reshaping the diagnostic and therapeutic landscape of OSCC, and explore microbiome‐targeted combination therapies. Addressing these challenges will be pivotal to realising truly intelligent, personalised management and ultimately improving outcomes for OSCC patients.

## Graphical abstract

Schematic overview showing how microbiota‐induced epithelial dysbiosis initiates OSCC pathogenesis; multi‐omics sequencing paired with machine learning modelling constructs predictive biomarkers; the resultant high‐precision prediction tools support early tumour screening, lymph node metastasis risk evaluation and individualised targeted treatment for OSCC patients.

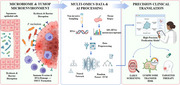

## 1 INTRODUCTION

Oral squamous cell carcinoma (OSCC), a common malignant tumour, arises from the stratified squamous epithelium of the oral mucosa, potentially affecting multiple anatomical sites, including the tongue, gingiva and buccal mucosa. In conjunction with oropharyngeal squamous cell carcinoma, it constitutes the predominant type of head and neck squamous cell carcinomas.^1^, ^2^ Current treatment involves a comprehensive strategy combining surgery, radiotherapy and chemotherapy^3^; however, the 5‐year overall survival rate—especially in those with advanced disease—remains unsatisfactory.^4^ Therapeutic resistance and tumour recurrence are still major challenges. Consequently, the development of more precise diagnostic tools and alternative therapeutic approaches has become critical for improving prognosis.^5^

In recent years, the role of the tumour microenvironment in OSCC progression and treatment response has attracted increasing attention,^6^ as it is now recognised as a key determinant of the biological behaviour of the tumour.^7^ The oral microbiome, a complex ecosystem colonising the oral cavity,^8^ is closely linked to host health and disease status.^9^, ^10^ In OSCC research, oral microbiome dysbiosis has been shown to correlate with risk factors such as smoking and alcohol use and to exhibit significant alterations in precancerous lesions, tumour tissues and saliva.^11^, ^12^, ^13^ Notably, the initiation and progression of OSCC may not depend on a single pathogen but rather on global functional dysregulation of the microbiome and the chronic microinflammatory environment it mediates, making it a potential prognostic marker and research target. Furthermore, antibiotics and other therapeutic interventions can profoundly impact the oral microbiome and, consequently, oral disease outcomes. For instance, antibiotic‐induced dysbiosis has been implicated in altering the progression of oral potentially malignant disorders. Therefore, understanding these effects is crucial for optimising treatment strategies in OSCC patients.^14^ Additionally, certain treatments that modulate the oral microbiota have shown promise in improving oral conditions, highlighting the need to integrate microbiomics into clinical decision‐making.^15^ These recent findings underscore the importance of antimicrobial and microbiome‑modulating therapies in the management of oral malignancies.

Artificial intelligence (AI) has achieved remarkable progress, driven by continuous improvements in computing capacity and the growing abundance of data.^16^ Machine learning (ML), a core branch of AI, enables automatic pattern extraction and prediction from data.^17^ In ML, artificial neural networks (ANNs) and their deep learning (DL) extensions with multiple hidden layers excel at high‑order feature learning and representation.^18^, ^19^, ^20^, ^21^, ^22^ Current DL‑based AI systems are widely used in cancer image analysis for automated tumour detection, feature characterisation and biomarker evaluation.^23^, ^24^, ^25^ Nevertheless, further clinical investigation is necessary to determine effective ways to leverage AI in microbiomics to improve the diagnosis and treatment of OSCC.

This article reviews the application of AI‑based microbiomics in OSCC to enhance diagnostic and therapeutic levels, ultimately improving patient prognosis.

## 2 DYSREGULATION OF THE ORAL MICROBIOME AND OSCC

Dysregulation of the oral microbiome is a key driver of the initiation and progression of oral cancer.^26^ This dysregulated state promotes tumourigenesis through a range of interconnected molecular and cellular mechanisms, including excessive microbial proliferation, abnormal regulation of host signalling pathways and the continuous release of bacterial toxins and virulence factors,^27^ further inducing and sustaining chronic inflammatory responses and carcinogenic DNA damage.^28^

**FIGURE 1:**
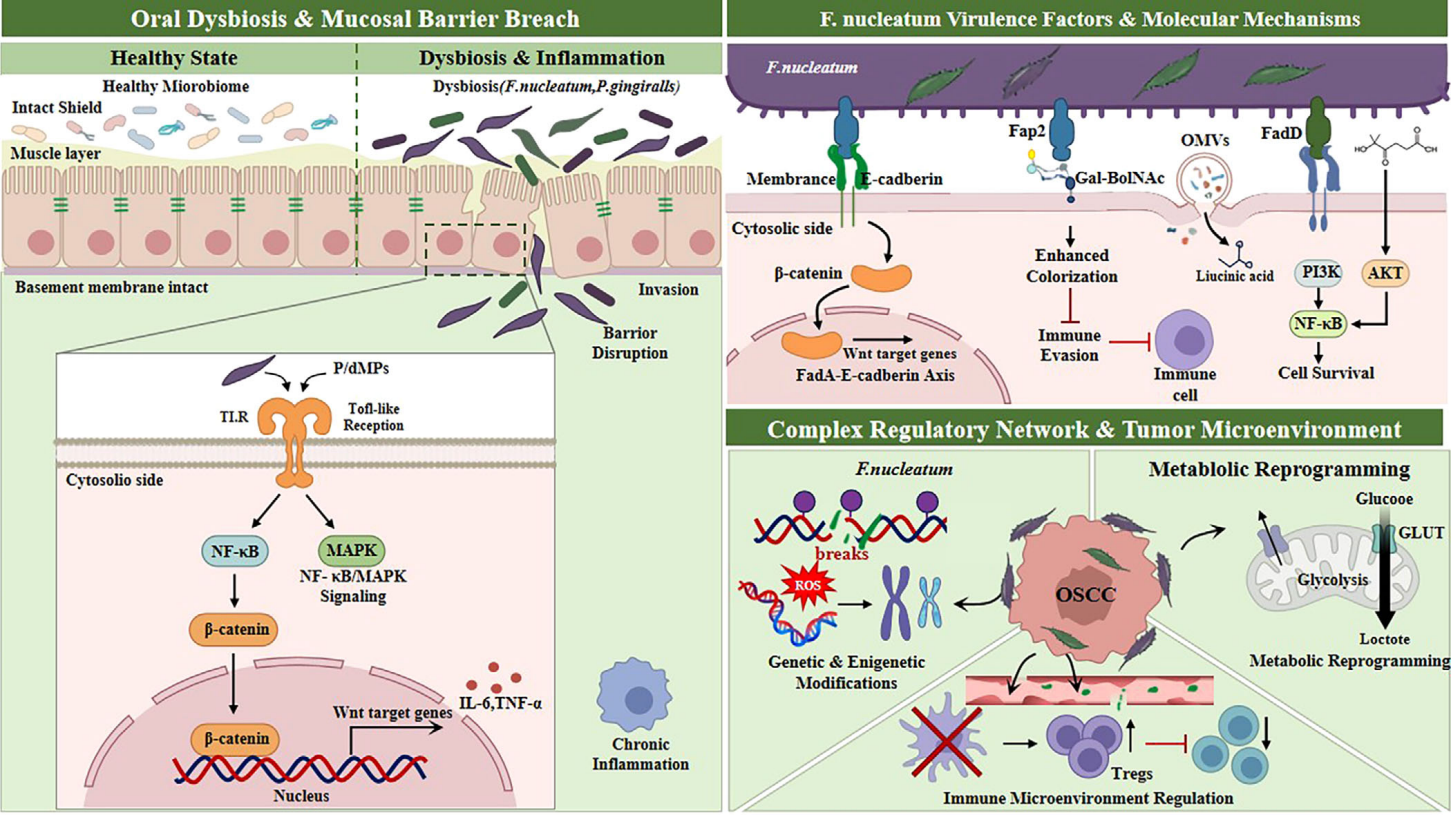
This composite figure illustrates the transition from oral homeostasis to dysbiosis and the molecular mechanisms by which F. nucleatum promotes OSCC progression. Healthy oral mucosa is characterised by an intact epithelial barrier, a structured basement membrane, and a balanced commensal microbiome. In contrast, dysbiosis with overgrowth of F. nucleatum and Porphyromonas gingivalis disrupts barrier integrity, triggers neutrophil infiltration, and induces chronic inflammation, facilitating bacterial translocation into subepithelial tissues. F. nucleatum components activate pattern‐recognition receptors (PRRs), including Toll‐like receptors (TLRs), on the cytosolic side of host cells, subsequently stimulating downstream NF‐κB and MAPK signalling cascades. This results in upregulated expression of pro‐inflammatory cytokines (e.g., IL‐6, TNF‐α), which sustain a pro‐carcinogenic inflammatory microenvironment. The upper panel details key virulence factors of F. nucleatum: Fap2 and FadA mediate adhesion to host cells via Gal‐GalNAc and E‐cadherin, respectively; FadD engages CD147 to activate PI3K‐AKT‐NF‐κB signalling; and outer membrane vesicles (OMVs) deliver bioactive molecules that modulate host responses. The lower panel depicts the complex regulatory network through which F. nucleatum shapes the tumour microenvironment, encompassing genetic and epigenetic modifications (e.g., Wnt/β–catenin activation), metabolic reprogramming (including enhanced glycolysis and the Warburg effect), and immune microenvironment regulation (e.g., suppression of effector T cells and induction of regulatory T cells). Collectively, these interconnected pathways drive chronic inflammation, immune evasion, and malignant progression in OSCC.

In health, the host and the resident microbiome are delicately balanced. However, microbial dysbiosis disrupts this homeostasis, particularly by activating pattern recognition receptors, most notably Toll‐like receptors^29^ and their downstream signalling cascades, impairing the structural and functional integrity of the mucosal barrier.^30^ Key signalling pathways in this process include the nuclear factor κu (NF‐κB) and mitogen‐activated protein kinase pathways. Mucosal barrier damage facilitates the invasion of bacteria and their metabolites into deeper tissues,^31^ further triggering persistent inflammatory responses with carcinogenic potential. Among the various microorganisms associated with oral cancer, *Fusobacterium nucleatum* and *Porphyromonas gingivalis* are particularly prominent^32^ (Figure 1).

### 2.1 Role of *F. nucleatum* in OSCC progression

*Fusobacterium nucleatum* directly promotes cancer development through multiple key factors it harbours, starting with its virulence factors, among which FadA (*Fusobacterium* adhesin A) is the most extensively studied. FadA specifically binds to E‐cadherin on the host cell surface and activates the downstream Wnt/β‐catenin signaling pathway,^33^ which plays a critical role in cell proliferation, differentiation and migration. Notably, the deletion of FadA significantly reduces the bacterium's carcinogenic capacity, preventing it from inducing colonic tumours in mice.^34^ Another virulence factor, Fap2, enhances bacterial colonisation at tumour sites by binding to the Gal‐GalNAc structure overexpressed on the surface of colorectal cancer cells, while simultaneously suppressing the host's immune response.^35^ Additionally, FadD binds to CD147 on colorectal cancer cells, activating the PI3K‐AKT‐NF‐κB signalling pathway to further promote tumour cell growth and survival^36^ (Figure 1).

In addition to these well‐characterised virulence factors, *F. nucleatum* also secretes various microbial metabolites, such as succinic acid and formic acid,^37^ which can cross the intestinal barrier, enter the host's circulatory system and affect the metabolic status and immune microenvironment of distant organs. Bacterial outer membrane vesicles constitute another important means of communication between *F. nucleatum* and host cells.^38^ These vesicles contain various bioactive molecules, including lipopolysaccharides, proteins and nucleic acids, which can be directly taken up by host cells to activate multiple intracellular signalling pathways.^39^ Finally, *F. nucleatum* can form symbiotic relationships with other pathogenic bacteria.^40^

Furthermore, *F. nucleatum* interaction with the host engages complex regulatory levels, including genetics, metabolism and immunity.

**FIGURE 2:**
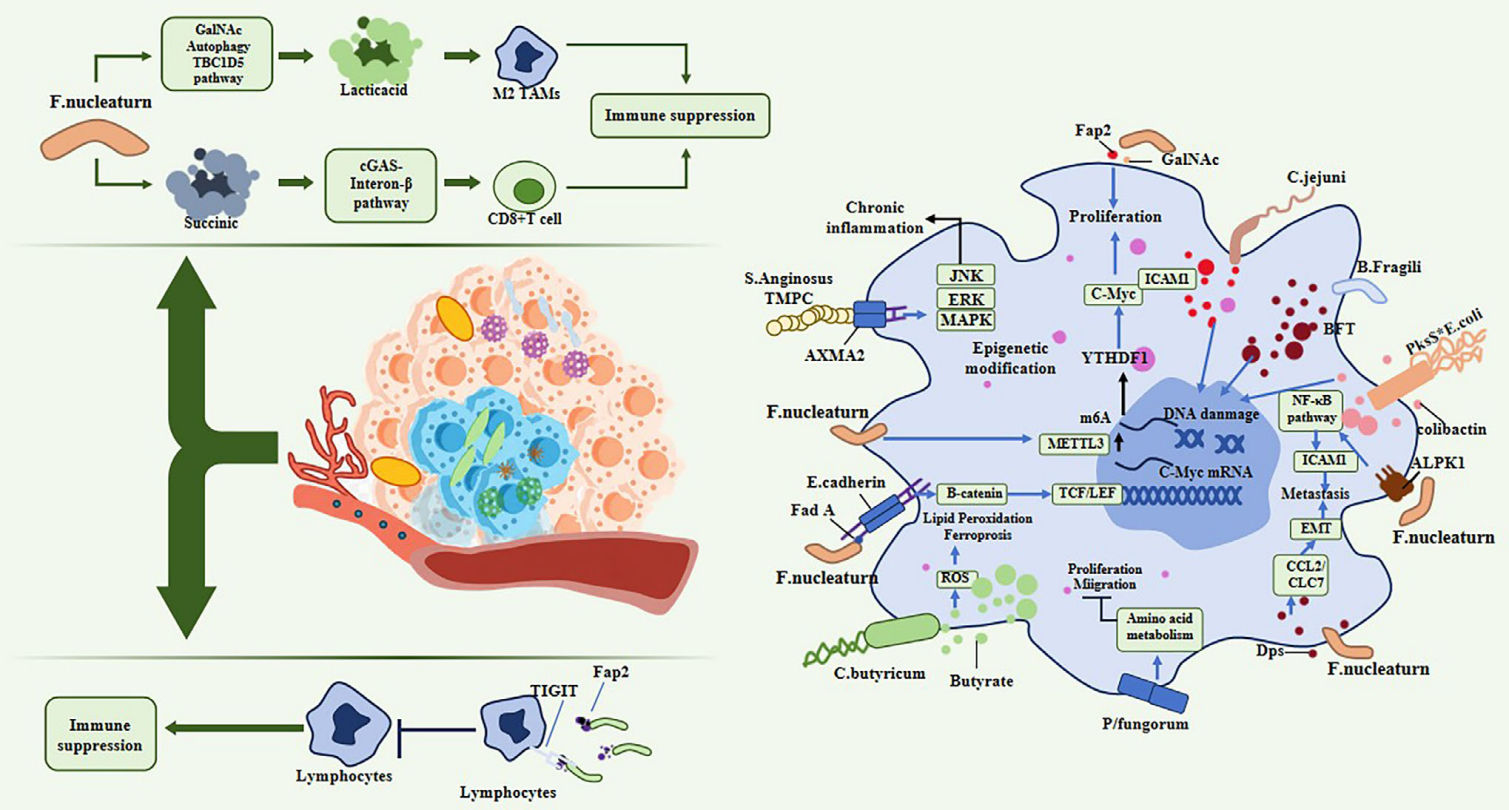
Multifaceted mechanisms of *F. nucleatum*: in modulating tumour microenvironment and immune evasion. This schematic illustrates the diverse pathways through which *F. nucleatum* influences tumour progression and immune regulation within the tumour microenvironment. The upper section highlights metabolic and immune‐modulatory mechanisms: *F. nucleatum*‐derived GalNAc promotes autophagy via TBC1D5; succinate activates the cGAS‐STING pathway, leading to interferon‐β production; and lactate contributes to immune suppression by polarising macrophages towards an M2 phenotype and inhibiting CD8+ T‐cell activity. The lower section depicts bacterial interactions and adhesion‐mediated effects: *F. nucleatum* synergises with *Streptococcus anginosus* to sustain chronic inflammation, while its adhesin Fap2 binds to GalNAc on tumour cells to enhance proliferation and upregulate ICAM1 and CD47. Additionally, *F. nucleatum* facilitates tumour cell invasion via FadA‐mediated E‐cadherin engagement and interacts with *Clostridium butyricum* to modulate butyrate levels. Key molecular players such as YTHDF1, METTL3 and various interleukins are indicated, reflecting the complex network of immune and epigenetic regulation driven by *F. nucleatum* in colorectal and other cancers.

1. Genetic and epigenetic levels: *F. nucleatum* infection increases the genomic instability of host cells, promoting gene mutations and chromosomal abnormalities.^41^ Meanwhile, the bacterium can also regulate host cell gene expression patterns through epigenetic modifications.
2. Metabolic reprogramming: *F. nucleatum* reshapes host cells’ metabolism through multiple mechanisms. For instance, it induces the Warburg effect, promoting glycolysis—even in oxygen‐rich environments.^42^ Tumour cells prefer glycolysis over mitochondrial oxidative phosphorylation for energy production, which provides the metabolic support necessary for rapidly proliferating tumour cells.^43^
3. Immune microenvironment regulation: *F. nucleatum* exhibits significant immunosuppressive properties. It interferes with the host's innate immune response by inhibiting dendritic cell maturation and function, thereby impeding antigen presentation.^44^ Additionally, the bacterium modulates T‐cell function, promoting the differentiation and expansion of regulatory T cells, while suppressing the activity of effector T cells and creating an immunosuppressive environment at the tumour site.^45^ Moreover, *F. nucleatum* impairs intestinal barrier function, increasing intestinal permeability and leading to endotoxemia and systemic inflammatory responses, which promote cancer progression and metastasis (Figure 2).

### 2.2 Role of other pathogenic microorganisms in OSCC progression

*Porphyromonas gingivalis*, a key periodontopathogen, produces a series of metabolites with carcinogenic potential, including reactive oxygen species (ROS).^46^ Among these, butyrate induces apoptosis in T cells and B lymphocytes, impairing the body's immune surveillance against tumour cells.^47^ On the other hand, oxygen‐free radicals directly damage genetic material by causing DNA double‐strand breaks, single‐strand breaks and base modifications.^48^ Acetaldehyde exacerbates DNA damage and promotes abnormal proliferation of epithelial cells, resulting in the malignant transformation of oral mucosal lesions.^49^

Similarly, *Prevotella intermedia* produces volatile sulfur compounds, such as hydrogen sulfide (H_2_S) and methyl mercaptan (CH_3_SH),^50^ which trigger oxidative stress in the periodontium and directly attack DNA structure. Hydrogen sulfide inhibits superoxide dismutase activity, impairing the cell's ability to scavenge superoxide‐free radicals and leading to the massive accumulation of ROS.^51^ Methyl mercaptan degrades type IV collagen in the basement membrane, compromising tissue structural integrity and facilitating tumour cell migration and invasion.^52^

Research has also shown that certain microbial infections can directly induce genomic instability. For example, *Mycoplasma parvum* infection increases γ‐H2AX levels (a marker of DNA double‐strand breaks) in tongue squamous cell carcinoma.^53^ Simultaneously, this bacterium downregulates the non‐homologous end joining repair protein Ku70 and the tumour suppressor protein p53, impairing the cell's ability to respond to DNA damage,^54^ exacerbating the accumulation of gene mutations and genomic instability, and further promoting the malignant progression of oral cancer.^55^

In summary, the oral microbiome drives the initiation, development and deterioration of oral cancer through complex mechanisms, including microbial dysbiosis, chronic inflammation and direct or indirect DNA damage. These mechanisms reveal the critical role of microorganisms in oral cancer and provide a theoretical basis for the future development of microbiome‐targeted intervention strategies, such as restoring microbial homeostasis and anti‐inflammatory therapy, with the potential to fundamentally reduce oral cancer risk.

## 3 AI‐BASED ORAL MICROBIOME ANALYSIS: METHODS AND MODELS FOR CANCER DETECTION APPLICATIONS

### 3.1 Data preprocessing: The cornerstone of AI‐driven microbiome analysis

The ability of AI to process complex, high‐dimensional data has established it as a powerful tool for extracting valid information in microbiome research. By analysing intricate datasets using computational algorithms,^56^ researchers can more accurately identify disease‐associated microbial signature patterns.^57^ Extensive research has demonstrated the widespread use of ML techniques in oral cancer detection and prognostic analysis.^58^ Notably, in recent years, AI has been successfully integrated into clinical healthcare practice, spanning diverse medical fields such as radiology, pathology and anesthesiology.^59^ The AI/Machine Learning Medical Device List released by the US Food and Drug Administration (FDA) shows that over 1000 relevant devices have been approved for commercial use, clearly indicating the accelerating adoption of AI in modern healthcare and its clinical value.^60^ As the FDA continues to facilitate the approval of innovative, safe and efficient medical devices,^61^ this list is growing rapidly. The increasing number of medical devices using AI and ML technologies, along with this expanding list of authorised devices, underscores the accelerated integration of AI into modern healthcare and the growing significance of its clinical value.^62^

The general framework of ML algorithms for oral microbiome‐based cancer detection comprises three stages: data preprocessing, training and inference.^63^ The training stage involves using feature engineering to extract features from labelled data and train the ML algorithm; the trained model is then applied in the inference stage to predict the cancer status of unseen data.^64^ For instance, Banaval's team developed a classifier based on microbial activity profiles using logistic regression with L2 regularisation. Studies by a team led by Granato and Amari, among others, used support vector machines (SVMs) or random forest models for feature selection and predictive modelling,^65^ which revealed associations between microbial composition and cancer prognosis.^66^ Learning‐based models can effectively analyse massive datasets, assisting researchers in developing tools for early diagnosis and precision medicine in oncology.^67^ A comprehensive framework is established by integrating taxonomic data, diversity metrics and predictive modelling to understand the role of microorganisms in tumour identification.^68^

Data preprocessing is a critical first step in developing an AI‐driven decision‐making system for microbiome‐based prediction of cancer.^69^ Proper preprocessing significantly enhances the performance and generalisation ability of ML models. Key challenges in microbiome data analysis include variations in sequencing depth, compositional effects^70^ and outliers, which necessitate the use of data transformation and standardisation techniques. Standardisation methods aim to rescale features to a standard range^71^ to prevent features with large values from dominating the learning process, with L1 and L2 normalisation among the most common approaches.^72^ Previous research has shown that combining standardised data with original taxonomic profiles can enhance the performance of classification algorithms. Another commonly used method is the logarithmic transformation to reduce feature dominance and mitigate the effects of outliers. Bayesian transformation techniques are effective solutions for addressing variations in sequencing depth and data sparsity.^73^

Unique analytical challenges exist in microbiome data preprocessing. In addition to the general preprocessing techniques described above, microbiome data present several inherent statistical and biological challenges that require dedicated handling. First, zero inflation is a frequent occurrence in metagenomic sequencing data, where a large proportion of microbial taxa are not observed across many samples due to limited sequencing depth, low biomass or true absence. Standard normalisation methods are inadequate for zero‐inflated data, as they can distort the underlying distribution. Advanced strategies have been proposed to address this challenge, including zero‐inflated negative binomial models, Bayesian imputation methods and the use of presence–absence indicators as separate features.^74^ Second, compositionality, that is, the fact that sequencing data reflect relative abundances rather than absolute quantities, violates the independence assumptions of many classical statistical methods. Simple correlation or differential abundance analyses performed on raw relative abundances can yield spurious associations. Log‐ratio transformations are widely recommended for converting compositional data into a Euclidean space suitable for ML.^75^ However, the CLR transformation is sensitive to zeros, necessitating prior zero‐handling steps that introduce additional complexity. Third, batch effects arising from different sequencing platforms, reagent lots or processing dates can significantly confound biological signals. Although general batch correction methods such as ComBat or limma exist, microbiome‐specific tools such as MMUPHin or PLS‐batch have been developed to account for the unique distributional properties of microbial count data.^76^ Failing to properly correct for batch effects can lead models to learn technical artefacts rather than true disease‐associated microbial signatures, severely compromising cross‐cohort generalizability. Fourth, current preprocessing methods rarely explicitly model ecological interactions. Most feature selection methods treat microbial taxa as independent predictors, ignoring microbial communities’ function as integrated networks. Emerging approaches, such as incorporating correlation‐based network metrics as additional features or using graph‐regularised ML models, aim to embed ecological interaction structures into the learning framework.^77^, ^78^, ^79^ However, these methods are currently underutilised and present computational and interpretability challenges. In summary, addressing these microbiome‐specific issues, including zero inflation, compositionality, batch effects and ecological interactions, during the preprocessing stage is essential for building robust, generalisable, biologically meaningful AI models for oral cancer detection.

### 3.2 Architecture selection and trade‐offs of ML models in microbiome analysis

Selecting an appropriate ML model is a crucial step in AI‐driven microbiome research.^80^, ^81^ It directly determines the model's predictive performance, computational efficiency and generalisation to unseen data, and profoundly influences the reliability of research conclusions and the feasibility of clinical translation. With the number of features often far exceeding the number of samples, microbiome data exhibit typical high‐dimensional characteristics, accompanied by significant noise interference and complex inter‐feature interactions.^82^ These unique data properties require researchers to strike a careful balance between model interpretability, nonlinear processing capabilities and computational costs.^83^ Currently, a range of ML algorithms, from classic linear statistical models to cutting‐edge DL architectures, has been widely applied in microbiome research, with each method demonstrating unique advantages and inevitable limitations in specific application scenarios.^84^

Among the models currently available, logistic regression holds an important position in biomedical research due to its excellent interpretability.^85^ As a generalised linear model, logistic regression effectively performs basic tasks, such as host phenotypic feature screening, and allows researchers to directly quantify the contribution of each microbial feature to the prediction result, thanks to its clear linear decision boundary.^86^ This property is particularly valuable in studies aiming to identify disease‐related biomarkers. For example, researchers can intuitively identify specific microbial groups that are positively or negatively correlated with oral cancer risk by analysing the magnitude and direction of feature coefficients.^87^ However, the linear nature of logistic regression also constitutes its main limitation. Complex nonlinear interactions, such as competition for niches and synergistic symbiosis, occur within microbial communities^88^; these intricate relationships often exceed the capture range of linear models.^89^ Therefore, during the processing of highly complex microbiome data, the performance of logistic regression is usually surpassed by more complex models.^90^

The naive Bayes classifier, which also emphasises practicality like logistic regression,^91^ is constructed based on Bayes’ theorem, simplifying calculations and estimating class posterior probabilities by assuming conditional independence between features.^92^ Although this strong independence assumption is often violated in real microbial ecosystems due to prevalent synergistic or competitive relationships between different microbial species,^93^ surprisingly, the model still demonstrates satisfactory robustness and high computational efficiency in many practical applications.^94^ The model's polynomial variant performs excellently on tasks such as 16S rRNA gene sequence classification, particularly in text classification and sequence analysis, enabling rapid and effective identification and categorisation of microbial species.^95^

Another simple and efficient instance‐based algorithm, the k‐nearest neighbor (KNN) algorithm,^96^ adopts a completely different learning paradigm. Without requiring a complex parameter training process, this algorithm makes classification decisions directly based on the relative proximity of samples in the feature space, which is intuitive and has led to satisfactory results in applications such as cancer identification based on salivary microbiota.^97^ Notably, KNN's performance largely depends on the selection of distance metrics and the neighbourhood size setting; these hyperparameters need to be carefully adjusted based on the specific characteristics of the data.^98^

Ensemble learning methods and complex nonlinear models become particularly valuable when prioritising prediction accuracy over model interpretability.^99^ Among them, random forests, an outstanding representative of ensemble learning, significantly improve the robustness and accuracy of the model by constructing a large number of decision trees and integrating their predictions.^100^ Its core mechanism, ‘bagging’ (bootstrap aggregation), provides each tree with a slightly different training subset by randomly sampling with replacement from the training data, thereby increasing the base learners’ diversity. Meanwhile, the ‘random feature subspace’ method considers only a subset of randomly selected features during node splitting, effectively reducing model variance and mitigating the risk of overfitting.^101^ In addition, random forests provide a built‐in function for evaluating feature importance. Researchers can systematically identify key biomarkers that contribute most to classification using metrics such as Gini impurity reduction or permutation importance, making it an ideal tool for exploratory microbiome research.^102^

SVMs are another powerful class of nonlinear models,^103^ whose core idea is to map raw data into a high‐dimensional feature space using kernel tricks, thereby finding an optimal classification hyperplane with maximum margin. Commonly used radial basis function kernels can effectively capture complex data's nonlinear patterns and are widely applied in high‐dimensional classification tasks such as identifying cancer biomarkers.^104^ However, SVMs also face significant challenges in multi‐class classification and with ultra‐high‐dimensional data. On one hand, traditional binary‐class SVMs need to be extended to multi‐class scenarios using strategies such as ‘one‐vs‐one’ or ‘one‐vs‐rest’, which significantly increases computational complexity^105^; on the other hand, high feature dimensionality with limited sample sizes predisposes models to overfitting, and the computational and storage costs of the kernel matrix rise sharply, limiting the applicability of SVMs in some large‐scale microbiome studies.^106^ ANNs provide the most powerful modelling tool currently available to address the extremely complex, nonlinear patterns that may exist in microbial systems.^107^ Inspired by biological neural networks, these DL models can automatically learn from raw data and extract discriminative hierarchical feature representations by combining cascaded connections of multi‐layer neurons and nonlinear activation functions.^108^ This feature‐learning capability of deep neural networks makes them particularly suitable for processing raw microbiome data that have not undergone extensive manual feature engineering.^109^ However, this strong expressive ability is achieved at the cost of model interpretability—the decision‐making process of neural networks is often regarded as an unexplainable ‘black box’, which limits their application to some extent in studies requiring clear explanations of biological mechanisms.^110^ In addition, deep neural networks typically require massive amounts of labelled data for training to avoid overfitting; however, in many microbiome studies, it is often difficult to obtain a large number of high‐quality labelled samples, posing one of the main obstacles to the widespread application of DL in this field.^111^

In addition to the above‐mentioned traditional architectures, two emerging AI paradigms are increasingly shaping the future of microbiome research. First, self‐supervised learning (SSL) has recently become popular as a powerful approach to use large‐scale unlabelled microbiome data. Unlike supervised methods that rely on costly labelled samples, SSL constructs pretext tasks, such as masked prediction, contrastive learning or reconstruction of microbial abundance profiles, to learn rich, generalisable feature representations from unlabelled data.^112^ For instance, contrastive learning frameworks can be designed to pull together augmented views of the same microbiome sample while pushing apart different samples, enabling models to capture intrinsic community structures without phenotypic labels. These learned representations can then be fine‐tuned on small, labelled datasets for downstream tasks such as classifying OSCC or identifying biomarkers.^113^ Given the high cost of obtaining clinically annotated microbiome data, SSL holds particular promise for oral microbiome research, where large‐scale shotgun metagenomic and 16S rRNA sequencing data are increasingly available but remain underutilised due to lack of labels. Second, explainable AI (XAI) techniques bridge high‐performance black‐box models and clinically actionable insights. Although traditional interpretable models like logistic regression offer transparency, they often fail to capture complex nonlinear interactions. XAI methods, including SHapley Additive exPlanations, Local Interpretable Model‐agnostic Explanations, attention mechanisms and layer‐wise relevance propagation, can be applied post hoc to complex models such as random forests, SVMs or deep neural networks.^114^ These techniques quantify each microbial feature's contribution to an individual prediction, enabling researchers to identify which specific bacterial taxa or functional genes drive the model's decision. Regarding OSCC, XAI can help validate whether AI‐predicted biomarkers align with known biological mechanisms or uncover novel, unexpected associations.^115^ Importantly, the integration of SSL and XAI is not mutually exclusive: SSL pre‐trains robust feature extractors from unlabelled data, while XAI applied to the fine‐tuned model generates interpretable outputs that address both data scarcity and ‘black‐box’ concerns simultaneously.

**FIGURE 3:**
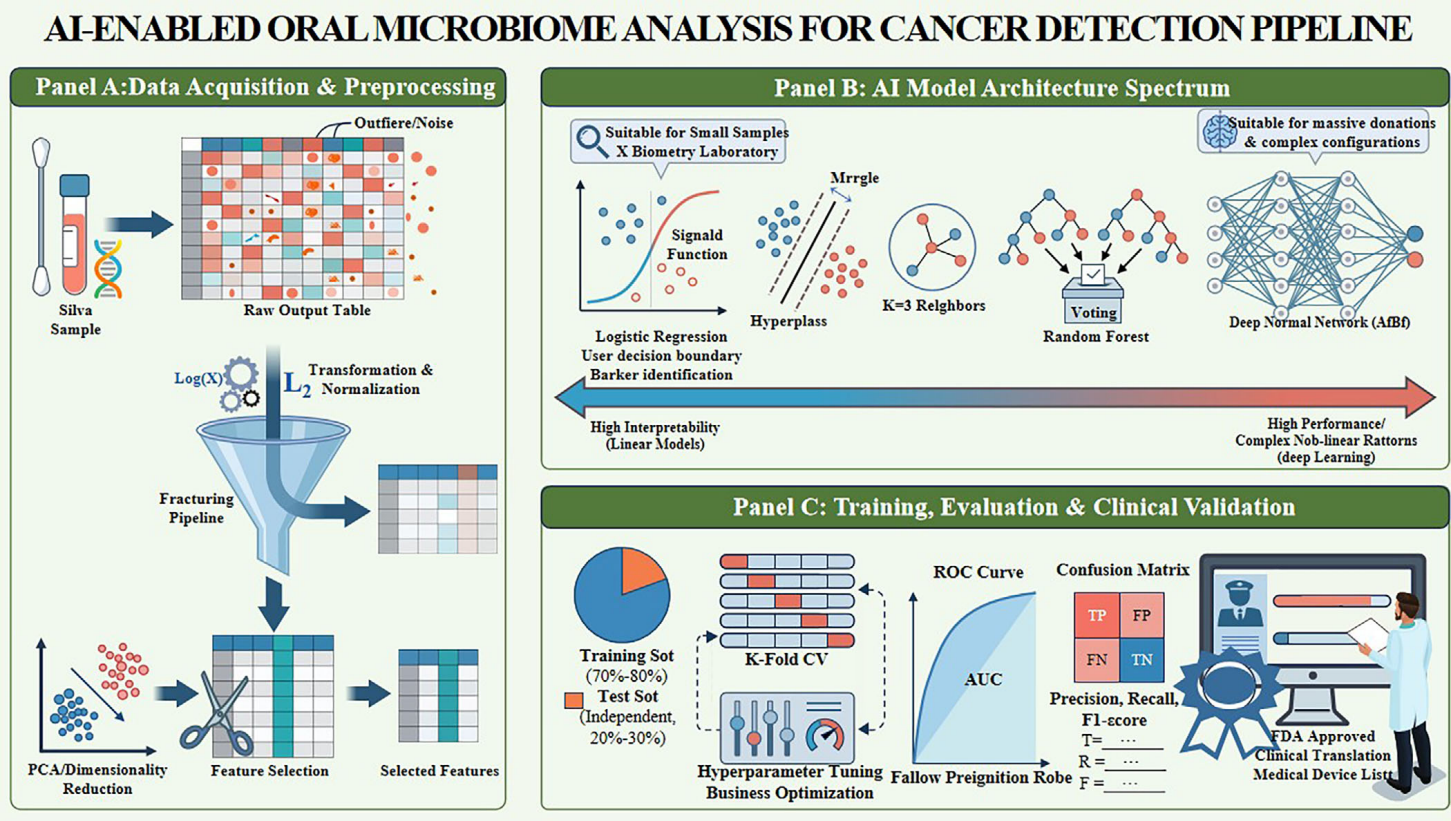
An artificial intelligence (AI)‐enabled pipeline for oral microbiome analysis in cancer detection. This figure illustrates an integrated pipeline for cancer detection through oral microbiome analysis leveraging AI. Panel A depicts the data acquisition and preprocessing steps. Starting with a saliva sample, a raw operational taxonomic unit table is generated, which is characterised by high‐dimensional data and sequencing depth variations. This raw data undergo a series of preprocessing steps including transformation and normalisation using methods like Log(x) and L2 normalisation, followed by filtering to reduce noise and outliers. Principal component analysis (PCA) and other dimensionality reduction techniques are then applied, along with feature selection (using Lasso, RF, random forest) to identify microbial signatures. Panel B presents the spectrum of AI model architectures suitable for this task. Logistic regression, a linear model with high interpretability, is suitable for small samples. Support vector machines/regression k‐nearest neighbors (SVM/R/KNNs) offer a balance. Random forest, an ensemble method, provides feature importance. Deep neural networks (ANN), on the other hand, are designed for massive datasets and complex patterns but have lower interpretability. Panel C details the training, evaluation, and clinical validation processes. The dataset is split into a training set (70%–80%) and a test set (20%–30%). K‐fold cross‐validation is employed during training, along with hyperparameter tuning using Bayesian optimisation. Performance metrics such as those from the receiver operating characteristic (ROC) curve (including area under the curve, AUC) and precision, recall, F1‐score from the confusion matrix are used for evaluation. Finally, the model undergoes clinical validation, with the potential for inclusion in the FDA‐approved clinical decision support medical device list.

In summary, no ‘one‐size‐fits‐all’ model exists for all research scenarios in the complex field of microbiome analysis.^116^ Model selection is essentially a multi‐purpose optimisation process, from logistic regression (pursuing high interpretability) to random forest (balancing predictive performance and partial interpretability) and from deep neural networks (dedicated to capturing extremely complex patterns) to emerging paradigms like SSL and XAI, which require precise matching with research objectives, data characteristics and available computational resources. In practical research, a hierarchical strategy is often adopted: First, simple models are used to establish baseline performance and yield preliminary biomarker clues, followed by gradually introducing more complex models to improve prediction accuracy; finally, the most reliable research conclusions are obtained through model comparison and ensemble learning. To make strategic choices that best meet research needs with this complex decision‐making process, researchers must be familiar with the theoretical basis of various models and deeply understand their specific trade‐offs in microbiome data analysis. Continuous development of ML technology, especially the rise of SSL, graph neural networks, meta‐learning and XAI in bioinformatics, will further expand the model selection space for future microbiome research and provide more powerful analytical tools to unravel the complex relationships between the microbiome and human health (Figure 3).

### 3.3 Evaluation techniques and validation strategies

The evaluation and validation of ML models are core steps in microbiomics research to ensure the reliability and reproducibility of research outcomes. The design and performance of a model are closely related to data characteristics, including the diversity of microbiome data, sequencing quality, sample size and specific research backgrounds and application scenarios.^117^ Researchers need to strike a balance across multiple dimensions during the model selection process: first, between interpretability and predictive performance.^118^

Models with high interpretability offer distinct advantages in studies that require an in‐depth understanding of the mechanisms linking microbial features to cancer development.^119^ For example, logistic regression models can clearly demonstrate the contribution of different microbial features to prediction results through interpretable coefficient weights; furthermore, decision tree models provide researchers with an intuitive ranking of feature importance via explicit decision rule paths.^120^ However, complex models like ANNs and gradient‐boosted machines often deliver superior performance in application scenarios where predictive accuracy is prioritised, such as early cancer screening, despite their ‘black‐box’ nature, which makes it difficult to understand their internal decision‐making mechanisms.^121^ When research prioritises performance and interpretability, ensemble learning methods like random forests offer an excellent compromise; they maintain high predictive performance while providing a degree of interpretability through feature importance analysis and the interpretation of individual decision trees.^122^

In addition to the balance between model performance and interpretability, computational cost and data availability must also be considered key factors in model selection.^123^ Regarding computational resources, significant differences exist between different model architectures: ANNs, especially deep neural networks with multiple hidden layers, require extensive matrix operations and gradient optimisation during training, which requires high computational hardware; the computational complexity of SVMs increases significantly with the number of support vectors when processing large‐scale datasets. In contrast, traditional models, such as logistic regression and decision trees, are associated with much lower computational costs.^124^

Data availability is an equally critical constraint on model selection. In microbiome research, high‐quality, labelled datasets are often limited in scale and costly.^125^ This practical limitation makes researchers more inclined to select algorithms that perform robustly on small‐sample data, such as logistic regression and SVMs, rather than DL models that require massive amounts of training data.^126^ Random forests, with anti‐overfitting properties from ensemble learning, and SVMs, which perform well on small‐sample, high‐dimensional data, have become the most commonly used modelling methods in microbiome research.^127^

The evaluation and validation of models require a systematic technical workflow to ensure the reliability of the results.^128^ The primary principle is to distinctly separate training and test data: A fully independent test set must be reserved to evaluate the model's generalisability, as this is the ultimate criterion for determining whether a model can be applied to real‐world scenarios.^129^ Building on this, cross‐validation techniques provide a more robust model evaluation scheme, particularly in stratified k‐fold cross‐validation. This method divides the original data into k mutually exclusive subsets and uses k − 1 subsets for training in each round and the remaining subset for validation through k rounds of iterative training and validation.^130^ Finally, the average of the k validation results is considered an estimate of the model's performance. In microbiome data, adopting a patient‐level stratification strategy is particularly important, as it ensures that each fold contains a representative distribution of patient characteristics, avoiding evaluation biases introduced by data partitioning.^131^

Hyperparameter tuning is a critical step in the model optimisation process; it requires systematic exploration of the hyperparameter space to find the optimal configuration.^132^ Different search strategies vary in computational efficiency and optimisation capability, ranging from traditional grid search and random search to more efficient and advanced methods such as Bayesian optimisation. Methods such as sequential model‐based Bayesian optimisation can typically find better hyperparameter configurations with fewer evaluations than traditional methods, especially when dealing with high‐dimensional hyperparameter spaces.^133^

A multidimensional indicator system should be established to quantitatively evaluate model performance and fully reflect the model's predictive ability.^134^ Accuracy, as the most intuitive indicator, reflects the overall correctness of the model's predictions but can be misleading in scenarios with class imbalance, a common issue in microbiome data.^135^ For example, in screening tasks where cancer‐positive samples are scarce, a model might achieve high accuracy by simply predicting all samples as negative; however, this completely ignores the core need to identify positive samples.^136^ Therefore, the F1 score is a more appropriate evaluation metric: It comprehensively considers the model's ability to identify positive samples through the harmonic mean of precision and recall.^137^ Precision measures the proportion of correctly predicted positive samples, reflecting the model's ability to avoid false positives; recall measures the proportion of all the true positive samples identified by the model, reflecting the model's ability to avoid false negatives.^138^ In scenarios such as the early diagnosis of oral cancer, high recall means fewer missed cases, while high precision means fewer false‐positive results.^139^ Researchers must balance these two metrics based on specific clinical application requirements. In addition to these basic metrics, comprehensive indicators such as the area under the receiver operating characteristic curve (AUC‐ROC) and the area under the precision‐recall curve (AUC‐PR) also play significant roles in model evaluation in microbiome research, providing a solid basis for model selection across different application scenarios.^140^

## 4 AI‐ASSISTED EXPLORATION OF THE LINK BETWEEN THE ORAL MICROBIOME AND CANCER

In recent years, AI has demonstrated great potential in unravelling the association between the oral microbiome and cancer, particularly OSCC.^3^ By developing diverse algorithmic models, researchers have explored in depth the connections between microbial features and cancer initiation and progression, yielding innovative insights for early cancer diagnosis and biomarker screening.^112^ Several studies have shown that different AI models exhibit distinct advantages in analysing oral microbiome data. Among these, the random forest algorithm is prominent as one of the most frequently used and high‐performing models, thanks to its ability to handle high‐dimensional data and resist overfitting.^68^ In this context, He et al. systematically evaluated the performance of classifiers such as random forest, SVM, ANN, and naive Bayes using oral microbiome data.^46^ They reported that random forest significantly outperformed other models in predicting OSCC risk. By optimising the feature combination of 20 specific bacterial genera, the model achieved an AUC of .99, surpassing traditional microbial dysbiosis indices and highlighting the precision of targeted microbial biomarkers in risk prediction. This finding suggests that the specific composition of the microbial community may serve as a reliable ‘molecular fingerprint’ for personalised assessment of cancer risk.

In addition to model selection, sample source diversity also influences the exploration of diagnostic efficacy. Lei et al. innovatively collected microbiome data from saliva, subgingival plaque, tumour surfaces, healthy oral mucosa and tumour tissues^51^ and developed two random forest models: one integrating information from all samples (accuracy: 98.17%) and a simplified model based solely on saliva (accuracy: 95.70%). External validation confirmed both models’ high accuracy in practical applications (96.67% and 93.58%y). This study verified the feasibility of minimally invasive sampling and identified potential associations between specific bacterial genera (e.g., *Actinomyces* and *Fusobacterium*) and OSCC via correlation analysis, laying the foundation for the development of non‐invasive diagnostic technologies.

Gao et al. focused on the tissue level.^56^ By comparing 16S rRNA sequencing data of cancerous and healthy tissues, they found that functional changes in the microbiome, such as abnormal amino acid metabolism and dysregulated glucose consumption, highly correlated with the pathological mechanisms of OSCC. They further screened 12 bacterial genera to distinguish cancerous tissues from adjacent non‐cancerous tissues, providing a functional perspective on the interaction between microbes and cancer.

Saba et al. focused on the impact of environmental factors. By analysing the oral microbiomes of smokeless tobacco users, they found that these users’ microbiomes were similar to those of OSCC patients, and specific microbes (such as Prevotella and Bacteroides) showed potential associations with tobacco exposure and cancer risk. This underscores the need to incorporate environmental covariates into microbiome research. Collectively, these studies demonstrated that ML can identify key microbial biomarkers and dissect the complex networks linking microbes to environmental exposure, tissue specificity and functional metabolism, offering a multidimensional perspective for precise cancer prevention and control.

Research into the link between the oral microbiome and cancer shows that AI applications extend beyond the construction of diagnostic models to cutting‐edge fields such as biomarker screening, metastasis risk prediction^98^ and cross‐cancer classification, thereby enhancing in‐depth understanding of cancer pathogenesis. To address the critical clinical issue of OSCC lymph node metastasis, Shao et al. analysed salivary samples from 54 patients using a random forest classifier.^90^ They reported significant differences in microbial diversity between the metastatic and non‐metastatic groups, with higher abundance of *Prevotella* and *Bifidobacterium* in patients with metastatic cancer. At the same time, the signal peptidase II‐related pathway was more active in patients without metastasis. This finding provides potential microbial indicators for assessing cancer progression risk and suggests that the microbial community may influence tumour invasiveness by regulating host signalling pathways.

Meanwhile, the Banavar team explored the application value of salivary samples in early cancer detection using a logistic regression model. A model built using metatranscriptomic data achieved an AUC of .9 for Stage I OSCC, with a sensitivity of 92.3% and specificity of 97.9%.^121^ After optimisation, the saliva detection technology underwent large‐scale validation (945 training samples plus 230 independent samples), maintaining a specificity and sensitivity of 94%/90% for OSCC and 84.2% for oropharyngeal cancer. This confirms saliva's reliability as a non‐invasive detection tool and highlights the synergistic diagnostic advantage of combining human mRNA with microbial biomarkers. This multi‐omics integration is expected to overcome the shortcomings of single biomarkers and improve the comprehensiveness and accuracy of detection.

**FIGURE 4:**
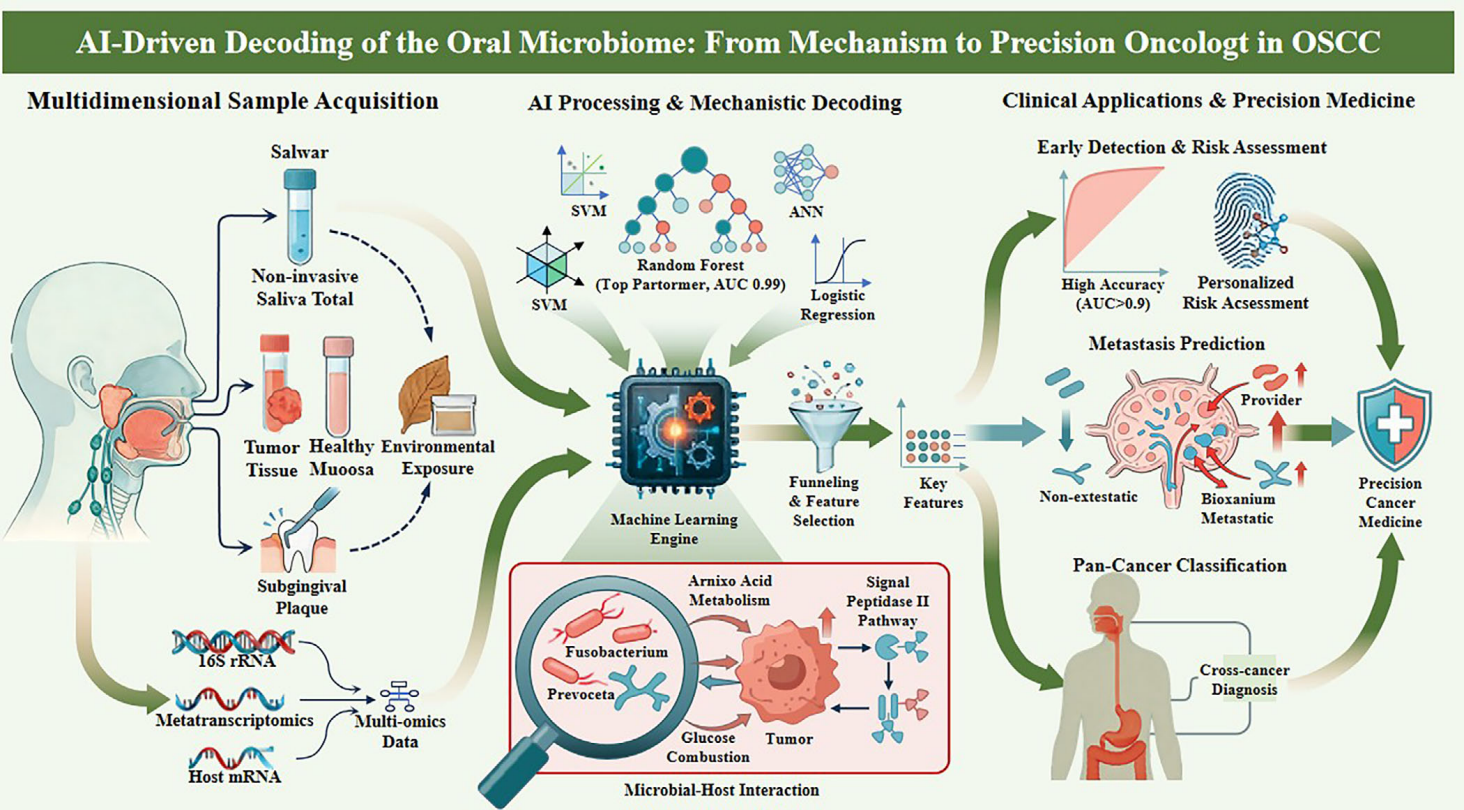
AI‐empowered deciphering of the oral microbiome: unveiling mechanisms and advancing precision oncology in oral squamous cell carcinoma (OSCC). This schematic diagram illustrates the comprehensive workflow from sample acquisition to clinical applications in the context of AI‐driven oral microbiome research for OSCC. In the ‘Multidimensional Sample Acquisition’ section, various types of samples such as saliva/swab, tumour tissue, healthy mucosa, environmental exposure samples and subgingival plaque are collected. These samples are then subjected to multi‐omics analyses including 16S rRNA, metatranscriptomics and host mRNA sequencing to generate multi‐omics data. The ‘AI Processing & Mechanistic Decoding’ part showcases the use of machine‐learning algorithms like SVM, random forest, artificial neural network (ANN) and logistic regression for processing the multi‐omics data. Feature selection is carried out to funnel and identify key features, which are then used to understand microbial–host interactions, such as amino acid metabolism and signal peptidase II pathway related to the tumour microenvironment. Finally, in the ‘Clinical Applications & Precision Medicine’ segment, the clinical implications of the research are highlighted, including early detection and risk assessment with high accuracy (AUC > .9), metastasis prediction by distinguishing between non‐metastatic and metastatic states, and pan‐cancer classification for cross‐cancer diagnosis, all contributing to precision cancer medicine. This integrated approach demonstrates the potential of AI in decoding the oral microbiome for improved OSCC management.

More notably, the Freitas team expanded their research scope to include pan‐cancer studies.^141^ Using cancer microbiome profile data to train a random forest algorithm, they evaluated the microbiome's ability to classify five cancer types, including head and neck, colon and gastric cancers.^123^ Although distinguishing adjacent‐site cancers was challenging due to similar microbial environments, the model performed well in classifying head and neck, gastric and colon cancers (with an accuracy of >90% for colon cancer). This finding confirms the potential value of microbiome data for cross‐cancer diagnosis and reveals the profound influence of anatomical sites on microbial composition: The microenvironment of different organs may selectively enrich specific microbial communities, forming a unique ‘microbe‐organ’ association pattern. From a broader perspective, it can be concluded from these studies that the oral microbiome is not merely a ‘bystander’ in cancer but an ‘active participant’ in disease initiation and progression.^45^ By efficiently processing complex high‐dimensional data, ML can extract key features from massive microbial datasets, assist in identifying biomarkers with diagnostic or prognostic value and further dissect the dynamic interaction networks between microbes and host physiological/pathological processes.^53^ Future deep integration of multi‐omics data and ML algorithms, with validation in larger‐scale clinical cohorts, is expected to make the oral microbiome a crucial breakthrough in precision cancer medicine, from early non‐invasive screening to personalised treatment monitoring, providing new strategies for improving patient prognosis (Figure 4).

## 5 AI‐DRIVEN TECHNOLOGICAL INNOVATION AND CLINICAL TRANSLATION PROSPECTS FOR EARLY DETECTION OF OSCC

In recent years, the integration of AI and oral microbiomics has opened new horizons for the precise diagnosis of OSCC and for biomarker screening. Traditional OSCC diagnosis relies on tissue biopsy and pathological analysis, which are limited by high invasiveness, low sensitivity in early stages and poor accessibility. In contrast, AI significantly improves the efficiency of extracting key information from complex microbial signals by processing multimodal microbiome data^142^ (Table 1).

**TABLE 1:** Artificial intelligence (AI) applications in oral cancer microbiome research.

AI application	Target bacterial species/microbiome feature	Relevance to oral squamous cell carcinoma (OSCC; mechanisms/biomarkers/diagnostic value)	Methodological approach	Ref.
Machine‐learning (ML) microbial biomarker identification	*Fusobacterium nucleatum*↑	Promotes OSCC via NF‐κB activation & EMT; diagnostic marker	Random forest	^143^
Deep learning (DL) classification	*Porphyromonas gingivalis*↑	Induces inflammation and DNA damage, enhances invasion	Convolutional neural network on 16S rRNA	^144^
ML‐based metagenomic profiling	*Capnocytophaga* spp. ↑	Enriched in OSCC lesions; associated with dysbiosis	Gradient boosting	^145^
Risk prediction modelling	*Prevotella* spp.↑/*Streptococcus* spp.↓	Linked to OSCC progression and TME alteration	XGBoost	^146^
Multi‐omics Al biomarker discovery	*F. nucleatum* + *P. gingivalis* consortium	Synergistic carcinogenesis; immune suppression	LASSO + integrative Al	^147^
Al‐assisted early detection	Anaerobic pathogens ↑	Distinguish OSCC versus healthy	RF+ artificial neural network (ANN) ensemble	^148^
Network inference model	IL‐6/IL‐8‐associated bacteria	Microbiome‐immune axis predicts OSCC	Bayesian network	^149^
Treatment response prediction	Microbiome predicting PD‐1 therapy response	Modulates TME inflammation	DL	^150^
SVM‐based microbial signature mining	*Treponema denticola* ↑	Induces ECM degradation and invasiveness	SVM classifier	^151^
Al feature ranking	*Tannerella forsythia* ↑	Periodontal pathogen associated with OSCC	ReliefF+RF	^152^
ANN‐based saliva classifier	*Actinomyces* spp.↓	Loss of commensals linked to carcinogenesis	ANN	^153^
Deep metagenomic clustering	*Campylobacter rectus*	Elevates inflammatory cytokines	Deep autoencoder	^154^
ML‐driven functional microbiome analysis	Butyrate‐producing bacteria ↓	Loss of anti‐inflammatory function	Random forest regression	^155^
Feature extraction with SHapley Additive exPlanations (SHAP)	*Peptostreptococcus stomatis*↑	Known OSCC‐associated species	XGBoost+SHAP	^156^
NLP‐assisted literature mining	OSCC microbial signatures	Automated extraction of bacteria‐OSCC associations	NLP+BERT	^157^
Al‐guided microbial co‐occurrence network	Pathogen clusters ↑	Bacterial synergy in OSCC	Graph neural network	^158^
Metabolic pathway prediction	Nitrosamine‐producing bacteria↑	Induce DNA damage	ML metabolic modelling	^159^
Al for HPV‐microbiome interaction	HPV‐positive OSCC microbial patterns	Distinct microbial ecology	Multi‐omics AI	^160^
OSCC staging model	Microbial diversity ↓	Correlates with tumour stage	SVM regression	^161^
ML for leukoplakia‐OSCC transformation	*Fusobacterium* enrichment	Predicts malignant transformation	RF classifier	^162^
Al‐supported volatile biomarker inference	Bacteria‐derived metabolites	Non‐invasive OSCC screening	ML metabolomic	^163^
Reinforcement learning optimisation	Microbial biomarkers	Identifies minimal diagnostic signature	RL feature selection	^164^
ML on paired tissue‐saliva microbiome	Concordant pathogens	Microbiome translocation hypothesis	RF	^165^
Deep phenotyping via AI	Immune‐microbiome patterns	Links bacteria to TIL alterations	Multi‐modal DL	^166^
Cancer hallmark prediction	Oxidative stress‐related microbiota	Predicts OSCC molecular subtype	ML classifier	^167^
Al‐based peritumoural microbiome mapping	Dysbiosis at tumour margins	Facilitates local invasion	Self‐organising maps	^168^
ML detection of rare pathogens	*Solobacterium moorei*↑	Emerging OSCC‐linked bacteria	Isolation forest	^169^
Al for microbiome time‐series	Longitudinal dysbiosis	Tracks OSCC initiation	LSTM model	^170^
Microbiome‐metabolite integration	Short‐chain fatty acid pathways↓	Linked to immune suppression	Multi‐omics integration	^171^
Precision AI for OSCC stratification	Composite microbial signature	High diagnostic AUC	Ensemble ML	^172^

*Note*: This table summarises AI‐driven analyses of oral microbiome features in OSCC, highlighting their roles in diagnostics, biomarker discovery and therapeutic prediction.

Abbriavtions: cGAS‐STING, cyclic GMP‐AMP synthase stimulator of interferon genes pathway; ECM, extracellular matrix; EMT, epithelial mesenchymal transition; HPV, human papillomavirus; IL‐6/IL‐8, Interleukin‐6 / Interleukin‐8; LASSO, least absolute shrinkage and selection operator; NF‐κB, nuclear factor kappa‐light‐chain‐enhancer of activated B cells; RF, random forest; RF+ANN, random forest plus artificial neural network (combined model); TIL, tumour‐infiltrating lymphocytes; TNF‐α, tumour necrosis factor‐alpha; TME, tumour microenvironment; XGBoost, eXtreme gradient boosting.

Regarding biomarker discovery, multiple studies have analysed the association between the oral microbiome and OSCC using AI algorithms,^103^ revealing the diagnostic potential of specific microbial combinations. Zhou et al. integrated microbiome data from multiple sites using a random forest model. They found that a model based solely on saliva data could achieve 95.70% accuracy and identified bacterial genera associated with OSCC, such as *Actinomyces* and *Fusobacterium*, laying the groundwork for non‐invasive screening.^100^ He et al. further optimised the model; a random forest classifier trained on 20 specific bacterial genera achieved an AUC of .99 for predicting oral cancer risk, significantly outperforming traditional microbial dysbiosis indices and confirming the precision of targeted microbial biomarkers. Additionally, to address the critical clinical issue of OSCC lymph node metastasis, Yin et al. analysed salivary samples from 54 patients using random forests. They reported a significant increase in the abundance of *Prevotella* and *Bifidobacterium* in the metastatic group; at the same time, the signal peptidase II‐related pathway was more active in the non‐metastatic group. This suggests that the microbial community can be used for cancer diagnosis and reflects the invasive characteristics of tumours.^98^

The core advantage of AI technology lies in its ability to integrate multidimensional data. Saxena et al. analysed 196 oral microbial samples from healthy individuals and OSCC patients using a random forest model, revealing high similarity between the microbiomes of smokeless tobacco users and those of OSCC patients.^142^ In addition, they identified potential associations between genera such as *Prevotella* and *Bacteroides* and tobacco exposure and cancer risk, highlighting the supplementary value of interactions between environmental factors and the microbiome in diagnosis.^173^ The Freitas team trained a random forest classifier on cancer microbiome profile data to assess the microbiome's ability to classify five cancer types (including head and neck and colon cancers). The model performed remarkably well in head and neck cancers, further confirming the microbiome's role as a pan‐cancer biomarker. These studies expand the scope of OSCC biomarkers (ranging from single bacterial genera to functional pathways and environment‒microbe interactions) and, through AI's ‘data‐driven’ model, advance traditional empirical diagnosis towards precision and personalisation.^174^

In the development of early detection technologies, AI‐assisted microbiome analysis is gradually overcoming the temporal and spatial shortcomings of traditional technologies, driving OSCC screening towards non‐invasiveness and convenience.^175^ Currently, saliva—as a representative of ‘body liquids’—has become an ideal carrier for AI applications due to its ease of sampling, non‐invasiveness and ability to reflect both local oral and systemic microbial states. The Banavar team trained a logistic regression model using 945 salivary samples and validated it with 230 independent samples, achieving 94% specificity and 90% sensitivity for OSCC.^176^ After further optimisation, the combined detection sensitivity for OSCC and oropharyngeal cancer reached 90%, with 84.2% specificity, indicating that AI combined with microbial biomarkers can enable early screening tools approaching clinical application standards.

More notably, AI is promoting the integration of microbiome and multi‐omics data to build a more comprehensive diagnostic system.^177^ On the one hand, the interaction between oral microbial metabolites and host immune/metabolic pathways has been shown to participate in OSCC pathogenesis. AI can more accurately capture cancer‐related functional changes by integrating microbial composition and metabolomic data.^178^ On the other hand, AI models can dynamically adjust diagnostic thresholds to improve applicability across different populations by combining histopathological images or patient clinical parameters. For instance, when Cao et al. used a convolutional neural network to analyse pathological images of oral cancer tissues, AI achieved 89.3% accuracy in classifying abnormal epithelial hyperplasia. Further integration of microbiome characteristics in lesion areas is expected to enhance diagnostic efficacy.^179^

From a clinical translation perspective, AI‐assisted OSCC microbial detection technology has entered the validation phase. Most previous studies have built models based on retrospective data; in the future, prospective cohorts are required to verify the stability and generalisability of these models.^143^ Meanwhile, the integration of portable detection devices and AI algorithms is expected to create a closed loop of ‘home sampling‐cloud analysis‐instant feedback’ to significantly improve the accessibility of screening. In addition, ethical and data security issues require concurrent attention.

In summary, AI‐driven oral microbiome analysis provides a ‘low‐cost, high‐sensitivity’ technical pathway for the early detection of OSCC and, through multi‐omics integration and adaptation to clinical scenarios, transforming cancer prevention and control from ‘passive treatment’ to ‘active early warning’, with significant public health importance and application prospects.

## 6 FUTURE OUTLOOK

The integration of AI with oral microbiome data has provided novel insights into the precise diagnosis and treatment of OSCC. Using multimodal data and algorithms, AI assists in identifying OSCC‐associated bacterial biomarkers and elucidating functional interactions between microbial communities and the tumour microenvironment. Clinically, AI‐assisted models overcome the shortcomings of traditional invasive diagnostic methods, advancing non‐invasive screening and early warning systems. Integration with multi‐omics data further enhances diagnostic specificity, laying the groundwork for personalised treatment. However, in addition to static biomarker discovery, discussing the evolution of microbial biomarkers is critical given the dynamic nature of oral conditions. The composition and functional potential of the oral microbiome are evolving across a continuum from oral health to precancerous lesions to OSCC, as well as during treatment and recurrence.

Certain bacterial taxa may shift in relative abundance or functional activity depending on disease stage, inflammatory status, exposure to antibiotics, surgical intervention or radiotherapy. For example, early‐stage OSCC's highly discriminatory biomarkers may lose specificity in patients with advanced disease or post‐treatment dysbiosis. It is critical to understand this temporal and conditional evolution to develop dynamic monitoring strategies, predict malignant transformation of precancerous lesions and assess treatment response or recurrence risk. AI models incorporating longitudinal sampling and time‐aware algorithms could capture such evolutionary trajectories; however, this remains an underexplored area. The current literature largely relies on cross‐sectional data, limiting our ability to infer causal or progressive relationships between microbial shifts and the evolution of oral disease. Currently, several practical barriers impede clinical translation, including the lack of standardised protocols for oral microbiome sampling and sequencing, with high inter‐study variability and poor model generalisability. Other barriers include the ‘black‐box’ nature of many DL models, which limits clinician trust and regulatory acceptance, the scarcity of large‐scale, prospective, multi‐center validation studies and the substantial infrastructure, computational resources and training required for real‐world implementation that are often unavailable in resource‐limited settings. Additionally, unresolved regulatory approval, data privacy and reimbursement barriers hinder widespread adoption.

## 7 CONCLUSION

In conclusion, overcoming current barriers, particularly the lack of standardised protocols, poor model generalisability and the ‘black‐box’ nature of AI models, is essential for clinical translation of AI‑based oral microbiome analysis in OSCC. Future research must prioritise three main objectives: developing hybrid algorithms that incorporate causal inference to capture dynamic microbe‒tumour interactions, conducting longitudinal mechanistic studies to validate biomarker evolution across the natural history of oral conditions and advancing clinical translation through prospective multi‑center validation, portable AI‑integrated devices and microbiome‑targeted combination therapies. Achieving these goals will enable the realisation of intelligent, personalised diagnostic and treatment paradigms in this emerging field.

## AUTHOR CONTRIBUTIONS

**Rui Shi ， Yuan Zhi**: Study conception and design; data collection, writing—original draft. **Wen‐Hao Ren**: Data collection; writing—review and editing; and visualisation. **Shao‐Ming Li**: Data collection and visualisation. **Ke‐Qian Zhi**: Supervision and funding acquisition. **Ling Gao**: Study conception and design, supervision and funding acquisition. All authors approved the final manuscript to be published.

## CONFLICT OF INTEREST STATEMENT

The authors declare no conflicts of interest.

## DECLARATION OF GENERATIVE AI IN SCIENTIFIC WRITING

We have used AI for language polishing, and the author is responsible for all content in the article.

## Data Availability

The authors have nothing to report.
